# Understanding the molecular mechanism of leaf morphogenesis in vegetable crops conduces to breeding process

**DOI:** 10.3389/fpls.2022.971453

**Published:** 2022-12-08

**Authors:** Ning Hao, Jiajian Cao, Chunhua Wang, Yipeng Zhu, Yalin Du, Tao Wu

**Affiliations:** ^1^ College of Horticulture, Hunan Agricultural University, Changsha, China; ^2^ College of Horticulture and Landscape, Northeast Agricultural University, Harbin, China; ^3^ Key Laboratory for Evaluation and Utilization of Gene Resources of Horticultural Crops, Ministry of Agriculture and Rural Affairs of China, Changsha, China; ^4^ Engineering Research Center for Horticultural Crop Germplasm Creation and New Variety Breeding, Ministry of Education, Changsha, China; ^5^ Guiyang Productivity Promotion Center, Guiyang Science and Technology Bureau, Guiyang, China

**Keywords:** leaf morphogenesis, vegetable crops, gene identification, molecular assisted selection (MAS), genetic map

## Abstract

Leaf morphology can affect the development and yield of plants by regulating plant architecture and photosynthesis. Several factors can determine the final leaf morphology, including the leaf complexity, size, shape, and margin type, which suggests that leaf morphogenesis is a complex regulation network. The formation of diverse leaf morphology is precisely controlled by gene regulation on translation and transcription levels. To further reveal this, more and more genome data has been published for different kinds of vegetable crops and advanced genotyping approaches have also been applied to identify the causal genes for the target traits. Therefore, the studies on the molecular regulation of leaf morphogenesis in vegetable crops have also been largely improved. This review will summarize the progress on identified genes or regulatory mechanisms of leaf morphogenesis and development in vegetable crops. These identified markers can be applied for further molecular-assisted selection (MAS) in vegetable crops. Overall, the review will contribute to understanding the leaf morphology of different crops from the perspective of molecular regulation and shortening the breeding cycle for vegetable crops.

## Introduction

Since the plant leaf is an organ that grows from almost indistinguishable primordia into shapes that vary largely among genus, species, or even cultivars (Kierzkowski et al., 2019), there have been several classification methods according to different characteristics. For example, leaves can be divided into simple or complex according to the number of leaflets, lobbed or serrated according to the margin shape, and inward-curvature or outward-curvature according to the curvature direction of the leaf margin. Well-organized gene and hormone regulatory mechanisms are the prerequisites for the formation of leaf diversity.

At present, the molecular mechanisms of leaf development and morphogenesis have been well studied in the dicotyledons plant, *Arabidopsis*, and the monocotyledons plant, rice. *Arabidopsis* typically has simple and small serrated leaves and is thus the ideal plant to study leaf shape and margin formation. Previous studies have shown that leaf serration formation depends on the guidance of auxin maxima at their tips (Kawamura et al., 2010), and many proteins can regulate the leaf serrations of *Arabidopsis* by direct or indirect auxin process (Kong et al., 2019). Auxin biosynthesis-related proteins, YUCCA (YUC), indole-3-acetic acid carboxyl methyltransferase (IAMT1), AUXIN-RESISTANT 1 (AUX1), and PIN-FORMED 1 (PIN1) (Hay et al., 2006; Wenzel et al., 2007; Kim et al., 2011; Kasprzewska et al., 2015) can regulate the auxin maxima directly in the leaf tips for *Arabidopsis*. Also, the auxin and ASYMMETRIC LEAVES1 (AS1) signaling pathways converge to suppress the expression of a *KNOTTED1-like homeobox* (*KNOX*) gene, *BREVIPEDICELLUS* (*BP*), which plays a key role in generating the auxin maxima at the tips of leaf serrations (Hay et al., 2006). The mutation in *INDOLE-3-BUTYRIC ACID RESPONSE 5* (*IBR5*) can result in changed serrations due to the disturbed auxin reporters and increased cell area (Kong et al., 2019). Besides the auxin regulatory process, other factors like gibberellins (GAs), cytokinins (CK), and microRNAs were also involved in leaf margin formation in *Arabidopsis* (Du et al., 2018), and these results also provide a further reference for the knowledge of leaf morphogenesis.

Unlike *Arabidopsis*, the research on rice leaf morphogenesis mainly focuses on its spatial extension due to the importance of ideal plant architecture, which plays a significant role in photosynthetic efficiency and grain yield (Heath and Gregory, 1938). Therefore, using molecular genetic techniques to produce proper leaf morphology and improve the photosynthesis rate can balance the relationship with the grain sink, which will effectively achieve a high yield in rice. For leaf curvature, four types of genes can control leaf curvature direction and degree according to the different gene functions, including genes regulating the adaxial/abaxial side, cell development in the adaxial side, development of sclerenchyma in the abaxial side and abnormal cuticle development (Fu et al., 2019). As for the leaf size regulation in rice, cell division-related genes, such as *SEMI-ROLLED LEAF 2* (*SRL2*), *SHALLOT-LIKE 1* (*SLL1*/*RL9*), *CATION-CHLORIDE COTRANSPORTER* (*OsCCC1*), and *WUSCHEL-related homeobox 4* (*OsWOX4*) (Yan et al., 2006; Zhang et al., 2009; Kong et al., 2011; Liu et al., 2016; Yasui et al., 2018), transcription factors and cellulases *NARROW AND ROLLED LEAF 1* (*NAL2*/*NAL3*), *SLL1* (Hu et al., 2010; Zhang et al., 2014) genes related to auxin synthesis and metabolism, *NAL7*, *TRYPTOPHAN DEFICIENT DWARF* (*TDD1*), *CONSTITUTIVELY WILTED 1* (*OsCOW1*), *OsARF19* (Woo et al., 2007; Fujino et al., 2008; Sazuka et al., 2009; Zhang et al., 2016) have been cloned and some of these genes have been widely used in the rice molecular breeding to cultivate the proper architecture.

Besides the research on the model plants, *Arabidopsis* and rice, there is still a need for further understanding of the leaf development of other species which is essential to understand species-specific spatiotemporal activation of conserved processes in leaf patterning. Vegetable crops are the essential nutrient resources in our daily life so there is a demand for high-yield production and quality. Leaf size has a direct effect on the production of leafy vegetables, and leaf shape has a close relationship with fruit quality (Rowland et al., 2020). With the further discovery of high-quality genomes for vegetable crops (Hao et al., 2020), the genes regulating the leaf morphogenesis in vegetable crops have been clarified from the genome sequencing and re-sequencing data. With the highly efficient transgenic system in many vegetable crops, the functional confirmation for these identified genes also becomes more reliable. Therefore, high-density and accurate genetic maps have been constructed for many important traits in vegetable crops. In this review, the present molecular studies on leaf morphogenesis in vegetable crops are summarized which is conducive to the understanding of leaf development and contributes to molecular-assisted breeding.

## The typical leaf morphology in vegetable crops

The leaf has diverse types in natural plants and can be classified according to different principles. Among them, leaf types can be generally classified into two categories according to the leaf initiation: simple or compound leaf. A simple leaf consists of a single undivided blade and a compound leaf consists of multiple leaflets, organized in pinnate, palmate, or higher-ordered structures (Champagne and Sinha, 2004). In vegetable crops, tomatoes and peas have been used as typical materials to study the formation of compound leaves. But the formation pattern of compound leaves is still quite different between these two species. Tomato has a pinnate compound leaf which is formed by three stages of leaflets, including primary (I), secondary (II), and intercalary (Int), and the leaflets have dissected margins (Blein et al., 2010). Peas have classic genetic material and some studies have indicated that their leaves are formed by several pairs of proximal leaflets (lt), distal tendrils (tdl), and leaflet-like stipules (st) (DeMason and Chetty, 2011). The complex leaf development of peas is usually divided into five stages. The P0 stage is differentiated from the apical meristem to produce the complex leaf primordium, then in the late P1 stage the primordium appears, and in the P2 stage develops the leaflet primordium. Tendril primordium develops in the P3 stage and the P4 stage forms terminal tendrils, which contribute to climbing and supporting plant growth (Gourlay et al., 2000). Therefore, due to the largely differentiated compound leaf development processes of these vegetable crops, the study of these species can contribute to a more comprehensive understanding of the process and mechanism of compound leaf development.

In addition, there is a class of important leafy plants in vegetable crops, whose leaves as the main edible organs, leaf development will greatly affect the yield and consumer choice. The proper curvature time and position of head leaves are essential for the formation of leafy heads. Among them, leafy heads vegetables include Chinese cabbage (*Brassica rapa ssp. pekinensis*, syn.) (Ren et al., 2020), cabbage (*B. oleracea* var. *capitata*) (Alemán-Báez et al., 2022), and lettuce (*Lactuca sativa*) (An et al., 2022). Leaf curvature as well as the occurrence of the leafy head is a complex biological process and also a morphological response induced by environmental factors. These factors can control the differentiation of abaxial–adaxial polarity during leaf development (Nath et al., 2003). Meanwhile, the polarity regulation process is related to cell cycle activity, cell size, cell division speed, and cell differentiation direction (Nath et al., 2003). If the cell division and growth at the edge of the leaves are slower than in the middle region, they develop into cup-like leaves with a positive Gaussian curvature value, whereas the leaves develop saddle-like with a negative Gaussian curvature value and form a leafy head (Ren et al., 2020).

Therefore, leaf morphology in vegetable crops is distinct from that in *Arabidopsis thaliana* or monocotyledonous rice and maize, which provides the basis for studying leaf morphogenesis. According to the previous study on the model plants and the development of genome sequencing of various vegetable crops in recent years, some important markers linked with leaf morphogenesis have been identified in vegetable crops and some known genes with novel functions were also confirmed.

## The regulation mechanism of leaf complexity in vegetable crops

Plant leaves initiate from the flanks of the shoot apical meristem (SAM), which are regulated by the complex genetic network (Du et al., 2018) and precise hormone concentration (Shwartz et al., 2016). When the leaf initiates from SAM, it is divided into either a simple or compound leaf and this is determined by some related genes and multiple hormones. In some vegetable crops, especially compound leaf crops such as tomatoes and peas, the molecular and physiological mechanisms also have been clarified and summarized in **Table 1**.

**Table 1:** Genes are involved in leaf complexity in tomato and pea.

Gene	Species	Description	phenotype	References
*KNOX1*	tomato	KNOX family	Overexpression of *KNOX1* caused more complex leaf structure	Bharathan and Sinha, 2001
*PTS*/*TKD1*	tomato	KNOX family	1 bp deletion in *PTS*/*TKD1* increased leaf complexity	Kimura et al., 2008
*UNI*	pea	*LEAFY* transcription factors	*uni* mutants produced the simple leaf	Wang et al., 2008
*BIP*	tomato	BEL-LIKE Homeodomain gene	bip *mutant showed highly complex leaf phenotype in* *heirloom tomato*	Nakayama et al., 2021
*CRISPA*	pea	MYB transcription factors	*crispa* mutants showed ectopic stipules, narrow leaflets, and shortened petioles with excessive adaxial expansion	DeMason and Chetty, 2014
*GOB*	tomato	CUC subfamily protein	Primary leaflets of *gob* mutant are often fused, secondary leaflets and marginal serrations are absent	Blein et al., 2008; Berger et al., 2009; Ben-Gera and Ori, 2012
*c*	tomato	RAX1 protein	c mutant reduced leaf complexity	Busch et al., 2011
PIN1	tomato and pea	Auxin efflux protein	*pin1* mutants influenced leaf and leaflet initiation	Koenig et al., 2009; Zhou et al., 2011
*leafless*	tomato	*DORNRONSCHEN* protein	*leafless* (*lfs*) in tomato can’t produce leaves and leaflets	Capua and Eshed, 2017
IAA9	tomato	Aux/IAA family protein	*iaa9* mutants showed simplified leaves	Wang et al., 2005; Zhang et al., 2007
*ARF10*	tomato	Auxin response protein	The mutation in *ARF10* inhibited the blade and leaflets outgrowth in tomato	Ben-Gera et al., 2016; Damodharan et al., 2016; Lin et al., 2016
*AGO7*	tomato	ARGONAUTE family	Overexpression of *AGO7* resulted in increased leaflet numbers	Lin et al., 2016
*clausa*	tomato	MYB transcription factor	*clausa* mutants elaborated compound leaves	Avivi et al., 2000; Bar et al., 2016
*GA2ox4*	tomato	Gibberellin 2-oxidase	Overexpression of *GA2ox4* increased leaf complexity	Yanai et al., 2011
*PRO*	tomato	DELLA protein	*pro* mutant showed simplified leaves	Marti et al., 2007; Bassel et al., 2008; Jasinski et al., 2008

In complex and fine gene regulatory networks, the *class1 Knotted-like homeobox* (*KNOX1*) gene family is essential for meristem initiation and maintenance of differentiation (Bharathan and Sinha, 2001; Wang and Jiao, 2020). When *KNOX1* genes are continuously expressed, they will result in the formation of complex leaf structures in tomatoes (Bharathan and Sinha, 2001). Then, the increased expression level of *KNOX1* can produce a more complex leaf structure (Hay and Tsiantis, 2010). A large-scale linkage analysis among the accessions with different leaf complexity was performed, and 1 bp deletion from the *KNOX* family gene (*PTS*/*TKD1*) was co-separated with the increased leaf complexity phenotype (Kimura et al., 2008). Similarly, a *Potato*-*leaf* (*c*) mutant showed reduced leaf complexity in tomatoes compared with the wild type, the causal gene of the *c* mutant was identified by the comparative genomics and showed different alleles among cultivars, which encoded Regulator of Axillary Meristem1 (RAX 1) and participated in leaf complexity patterning (Busch et al., 2011). Recently, two different Homeobox genes in tomatoes, *BIPINNATA* (*BIP*) and *WUSCHEL RELATED HOMEOBOX 1* (*WOX1*), contribute to leaf complexity and leaflet development, respectively (Nakayama et al., 2021). Comparative genomics analysis among the wild type and domesticated tomato species revealed the *bip* mutation in heirloom tomatoes arose *de novo* during the domestication process (Nakayama et al., 2021). Therefore, such a locus identified from the natural population can be a reliable marker for the selection of tomato leaf morphology.

Unlike tomatoes, studies have found that peas belong to the legumes inverted repeat-lacking clade (IRLC) family of plants. This branch plant has a different compound leaf regulation mechanism from tomatoes and other legumes (Bar and Ori, 2015). To begin with, *KNOX1* is not expressed in the leaf primordia of peas, suggesting that *KNOX1* is not involved in compound leaf development in peas (Hofer et al., 2009). Instead, *UNIFOLIATA* (*UNI*) in peas encodes *LEAFY* (*LFY*)-type transcription factors and plays a similar role as *KNOX1* in tomatoes (Hofer et al., 1997). *UNI* is initially expressed in the complex leaf primordium when the leaflets have not differentiated. With the differentiation of the leaflet primordium, the expression level of *UNI* was enhanced in the newly produced leaflet primordium (Hofer et al., 1997). Pea *uni* mutants produced simple leaves which also indicated that *UNI* maintains the complex leaf development state in peas (Hofer et al., 1997). Therefore, the homologous gene of *LFY* in peas acts as a key regulator to control the formation of compound leaves.

Besides, there are some genes regulating the leaf complexity on the transcriptional level. *PHANTASTICA* (*SlPHAN*), containing an MYB-domain protein, can regulate leaflet formation in tomatoes (Kim et al., 2003a). The expression pattern indicated that *SlPHAN* was expressed throughout the entire adaxial side of the leaf primordium and formed pinnate compound leaves (Kim et al., 2003a). The increased expression level of *SlPHAN* also can result in a variety of leaf morphologies, from simple, ternate to compound leaves (Zoulias et al., 2012). Meanwhile, *SlPHAN* has a complex relationship with the *KNOX* gene family to regulate complex formation in tomatoes. *LeT6* and *SlPHAN* are expressed in the compound leaf and leaflet primordium (Kim et al., 2003b), but *SlPHAN* is down-regulated in overexpressed *LeT6* mutant, which indicates that *LeT6* is a negative regulator of *SlPHAN* (Kim et al., 2003b). There are other MYB transcription factors identified in peas. *CRISPA* is the ortholog of *PHAN* in peas, *the cri-1* (*CRISPA*) mutant exhibits ectopic stipules, narrow leaflets, and shortened petioles with excessive adaxial expansion (Tattersall et al., 2005). *CRI* suppresses *KNOX1* and *UNI* to maintain the normal development and expansion of leaves in peas (DeMason and Chetty, 2014). *GOBLET* (*GOB*) belongs to the CUP-SHAPED COTYLEDONS (CUC) subfamily, which can affect compound leaf patterning largely by its expression position, time, and level *(* Blein et al., 2008; Berger et al., 2009; Ben-Gera and Ori, 2012). *GOB* is negatively controlled by the MYB transcription factor *CLAUSA* (*CLUA*) and microRNA miR164, whose ectopic expression in tomatoes can result in extremely elaborate compound leaves and absent secondary leaflet initiation, respectively (Berger et al., 2009; Bar and Ori, 2015).

Precise hormone regulation is also involved in the formation of leaf complexity in vegetable crops. Firstly, the leaf development from the primordium requires the distribution of auxin in precise locations within a specific spatiotemporal developmental context (Koenig et al., 2009; Ben-Gera and Ori, 2012). The treatment of exogenous auxin or auxin transport inhibitors induces leaflet initiation or the simplification of leaf forms in peas and tomatoes, respectively (DeMason and Chawla, 2004; Koenig et al., 2009). Auxin activity maxima are also related to leaflet initiation. An auxin efflux protein PIN1 coincides with leaf and leaflet initiation in tomatoes and peas (Bai and DeMason, 2006; Koenig et al., 2009). The recombination-based mapping results showed that a severe auxin-defective tomato mutant *leafless* (*lfs*), which cannot produce leaves and leaflets, is due to the transient expression of *DORNRONSCHEN* (*DRN*) at incipient and young primordia overlapped with auxin response maxima (Capua and Eshed, 2017). Additionally, there are some auxin signaling and response proteins involved in the subsequent development of leaf dissection. The mutation in SlIAA9, an auxin response protein, exhibited simplified leaves and a similar phenotype in the tomato mutant *entire* (*e*), indicating that *IAA9* was the causal gene for the *e* mutant (Wang et al., 2005; Zhang et al., 2007). The expression level of *E/SlIAA9* has an effect on restricting lamina outgrowth between leaflets (Koenig et al., 2009; Ben-Gera and Ori, 2012). At the translation and post-translation level, E/SlIAA9 can interact with TRANSPORT INHIBITOR RESPONSE1 (SlTIR) and AUXIN F-BOX (SlAFB6), which are auxin receptors, and can be degraded by the ubiquitin 26S proteasome SCFTIR1/AFB under specific auxin level (Dharmasiri et al., 2005; Kepinski and Leyser, 2005). The auxin response protein family (ARFs) has a redundant function in leaf complexity and is always controlled by microRNAs (Ben-Gera et al., 2016; Wu et al., 2018). The repression of *SlARF10* by *SlmiR160* can inhibit the blade and leaflet outgrowth in tomatoes (Ben-Gera et al., 2016; Damodharan et al., 2016; Lin et al., 2016). Besides, the activity of ARF proteins is also negatively controlled by the trans-acting small interfering RNAs (ta-siRNA) and they are involved in leaf development among different plant species (Yan et al., 2010; Lin et al., 2016). In tomatoes, the tasiRNA-ARFs module was an ARGONAUTE (AGO)-dependent process. A wiry mutant with a simpler and narrow leaf in tomatoes is due to a mutation in *AGO7*. Conversely, the overexpression of *AGO7* in tomatoes altered the production of ta-siRNA and repression of ARFs activity (Yifhar et al., 2012; Lin et al., 2016). It is demonstrated among different species that gene regulation or exogenous auxin application will cause disrupted auxin distribution and affect leaf and leaflet initiation.

Besides auxin, GAs and CK are also important for leaf morphological formation in some vegetable crops. CK usually cooperates with auxin to regulate leaf initiation. Endogenous CK can increase the expression level of CK-related genes *ISOPENTENYL TRANSFERASE 7* (*IPT7*) and *CYTOKININ OXIDASE/DEHYDROGENASE3* (*CKX3*) in tomatoes and then change the leaf complexity (Shwartz et al., 2016). Both local applications of auxin to develop leaf primordia and mutations in *SlIAA9* can suppress the expression level of CK biosynthesis gene *IPT7* and the super-compound leaf formation, suggesting that CK regulation of dissected leaf morphogenesis requires a localized auxin response (Shwartz et al., 2016). An MYB transcription factor mutant *clausa* showed increased leaf complexity. CK signaling was enhanced by *CLAU*, thus uncovering an additional instance in which heightened CK signals result in increased complexity of the tomato leaves (Avivi et al., 2000; Bar et al., 2016). Similarly, the application of exogenous GA will lead the leaves to form a simplified leaf phenotype in tomatoes (Hay et al., 2002; Fleishon et al., 2011). Some GA-related genes are also involved in the formation of leaf complexity in tomatoes and peas. GA up-regulated the expression level of *UNI* to make the leaves more complex in peas (DeMason and Chetty, 2011). The overexpression of a GA-responsive gene *Gibberellin 2-oxidase* (*GA2ox4*) in tomatoes and peas caused increased leaf complexity (Yanai et al., 2011). The mutation of the homologous gene *PROCERA* (*PRO*) which encodes DELLA protein in tomatoes exhibits simplified leaves (Marti et al., 2007; Bassel et al., 2008; Jasinski et al., 2008). Besides, GA application or *clau pro* double mutants can repress the compound leaf phenotype of the *clau* mutant, failing to delimit the expression domain of *KNOXI*, then displaying elevated *KNOXI* expression range and increased leaf complexity (Hay et al., 2002).

## Genes related to leaf curvature in vegetable crops

**Figure 1:**
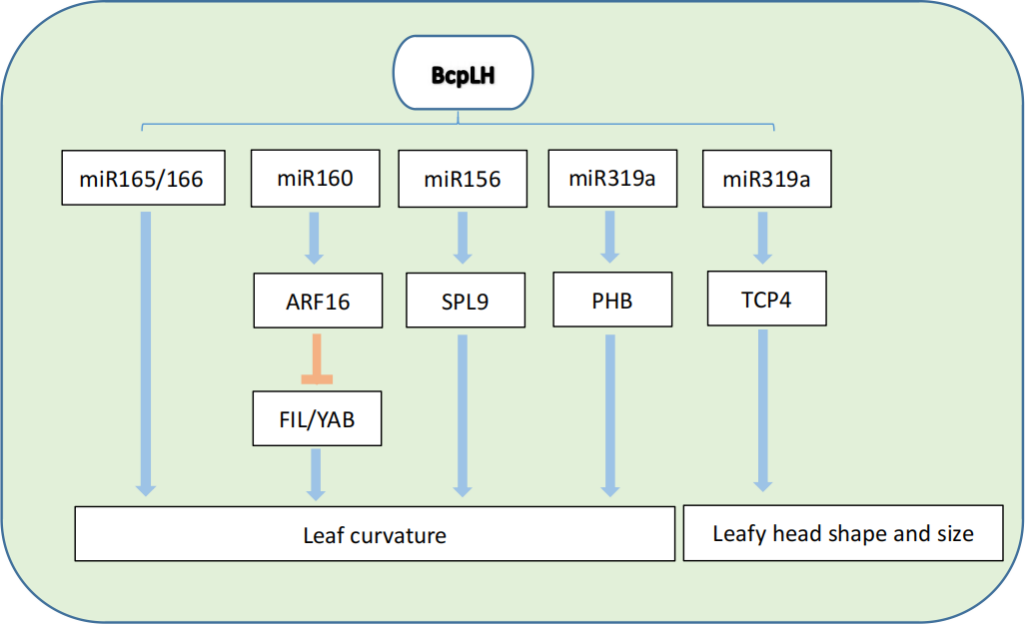
Genetic regulation of leafy head development in Chinese Cabbage.

The different models of leaf adaxial-abaxial patterning can influence the leaf initiation and following development process. At the initiation stage, the defective adaxial-abaxial patterning can influence the normal initiation from the SAM and, at the development stage, the adaxial-abaxial patterning can also result in the upward- or downward-curling leaf phenotype (Yamaguchi et al., 2012). Leaf curvature is the most important factor in leafy head formation and the occurrence of the leafy head is also regulated by several adaxial-abaxial related genes and environmental factors (Ito, 1957; Liang et al., 2016). Among them, two curvature-related genes have been cloned in Chinese cabbage, *Brassica rapa ssp. pekinensis LEAFY HEADS* (*BcpLH*) and *BcpLH2*, which may have a redundant function in leafy head formation (Yu et al., 2000; Ren et al., 2020). *BcpLH* is a close homolog of Arabidopsis HYPONASTIC LEAVES 1 (HYL1) in Chinese cabbage, which participates in miRNA biogenesis. *BcpLH* can control curvature development and leafy head formation in Chinese cabbage by coordinating with a set of microRNAs, including miR165/166, miR160, and miR319a to regulate the time of leaf curvature (**Figure 1**) (Ren et al., 2018; Ren et al., 2020). Conversely, these microRNAs can target some adaxial-determined genes and control the expression ratio to determine the degree and direction of leaf curvature. miR319a can positively regulate the abaxial-determined gene *PHABULOSA* (*PHB*), and *ARF16* is a target gene of miR160 which can negatively regulate adaxial-determined gene *FIL*/*YAB1* (Liu et al., 2011; Ren et al., 2018; Ren et al., 2020). *SQUAMOSA PROMOTER BINDING PROTEIN-LIKE 9* (*BrpSPL9*) was identified by the transgenic system and miR156 can regulate *BrpSPL9* to control leaf curvature, which has been considered to be a crucial gene for genetic improvement of Chinese cabbage (Wang et al., 2014). Using the recombinant inbred line (RILs) of heading and non-heading Chinese cabbage combined with QTL analysis, the miR319-targeted gene *TEOSINTE BRANCHED1/CYCLOIDIA/PCF* (*TCP4*) can regulate the shape and size of the leafy head by controlling the expression level of *TCP4* (**Figure 1**) (Yu et al., 2013; Mao et al., 2014). The other transcription factor *BcMYB101* was also identified to possibly participate in the development of curly leaves in non-heading pak choi (Hou et al., 2020).

In order to further investigate the molecular mechanism of leafy head formation, segregated populations were constructed using non-heading pak choi (PC-101) with Chinese cabbage, then a high-density DH-88 genetic map for leafy head formation-related QTLs was generated, which was located on the chromosomes A03, A05, and A08. These QTLs related to heading traits were identified to contribute the breeding selection in Chinese cabbage (Sun et al., 2018). Another recombination of pak choi (PC-101) and Chinese cabbage (CC-48) was also constructed and 22 QTLs for leafy head-related traits were discovered. Among them, five QTLs were located on chromosomes A04 and A05 linked with head leaf shape (Sun et al., 2021). In lettuce, two cultivars including the heading lettuce (PI536839) and the non-heading lettuce (PI344074) were used to generate a genetic population. After BSR-seq and complementation test, a homologous gene of *knotted 1* (*KN1*) in maize was the responsible gene for heading in lettuce. KN1 protein in lettuce (LsKN1) has conserved KNOX and homeobox domains (Yu et al., 2020). Besides the regulation at the genetic level, the plant hormones and environmental conditions are also critical for leaf curvature. Previous physiological experiments have confirmed that the formation of the leafy head of cabbage is due to the growth of the unbalanced distribution of auxin on both sides of the leafy head (Dong and Huang, 2018). External environmental factors can influence leafy head development including the photosynthesis period, temperature, and fertilizer application (Ito, 1957). Thus, the cooperation between genetic background and proper growth environment can promote the good growth of leafy vegetable crops.

## Genes related to leaf shape and size formation in vegetable crops

After leaf initiation from SAM, determinate cells are induced to change division patterns along three axes: proximal/distal, adaxial/abaxial, and medial/lateral. These affect the later cell division and extension, thus controlling the final leaf size and the formation of the leaf margin (Dkhar and Pareek, 2014). The complicated process of leaf shape and size also indicates they are quantitative traits and are controlled by multiple genes. Therefore, several QTLs or genes linked with leaf shape have been identified in vegetable crops by QTL mapping combined with other sequencing methods. For instance, two QTLs (*qLeaf_or-1*, *qLeaf_or-2*) with a high effect on leaf traits were mapped between two pea lines with *tendrilless* and *afila* leaf types and combined with specific locus amplified fragment sequencing (SLAF-seq), a high-density genetic map was constructed containing many SNP markers linked to leaf traits (Zheng et al., 2019). A comparative mapping and gene cloning approach among *bio*, *ele1* (larger leaf size), and *lath* (smaller leaf size) identified a KIX domain protein and an ortholog of *Arabidopsis* PEAPOD (PPD) which were involved in organ size determination in peas (Li et al., 2019). A WD40 repeat domain-containing protein was mapped to regulate the leaf size in cucumbers and the mutation site W264 in this protein was also conserved in the diverse natural cucumber population. Besides genetic analysis, the transcriptome analysis also provided evidence that *LL* might regulate or interact with some known organ size genes including *AP2-like ethylene-responsive transcription factor* (*ANT-like*), *GROWTH-REGULATING FACTOR* (*GRF-1*), *GRF-4*, *GIF-1*, *TCP4*, *TCP7*, and *TCP15* to regulate the leaf and lateral organ size together in cucumbers (Yang et al., 2018). The characteristics of the leaf margin and the underlying mechanisms also confer additional complexity which results in diverse leaf shapes. The main types of leaf margins are entire, serrated, or lobed. Recent genetic analysis and mapping of the genes related to the lobed-leaf trait in various vegetable crops showed that the lobed-leaf is controlled by a single dominant gene, *LOBED LEAF1* (*LL1*), in watermelons (*Citrullus lanatus* L.) (Wei et al., 2017), but by a single recessive gene in melons (*Cucumis melo* L.) (Gao et al., 2014). On the contrary, it was believed that the lobed leaf was a quantitative trait and it was speculated that four candidate genes were responsible in ornamental kale (Ren et al., 2019). Later, a fine mapping and BSA-seq result from two inbred lines with different leaf phenotypes in ornamental kale indicated the candidate gene for the feathered-leaved trait (BoFL) was located on chromosome C9 and co-segregated with a CAPS marker (CAPS4610) (Feng et al., 2020). Some known proteins performed a novel function in the leaf margin development of vegetable crops. The SNP in *CsPID* identified by MutMap^+^ analysis caused the palmate cucumber leaves to become round with a lobed margin (Zhang et al., 2018; Liu et al., 2019; Song et al., 2019). PINOID (PID) encodes a serine/threonine kinase and catalyzes the phosphorylation of auxin efflux transporter PIN to regulate the cellular auxin efflux, which has a function in inflorescence and root development in *Arabidopsis*, maize, and rice (Benjamins et al., 2003; McSteen et al., 2007; Morita and Kyozuka, 2007). But in cucumbers, *CsPID* not only participates in floral organ development but also regulates leaf margin development (Zhang et al., 2018; Liu et al., 2019; Song et al., 2019).

There are also other genes regulating the leaf shape and size formation in the transcriptional process. YABBY family genes have been reported to be involved in auxin flux and auxin response in *Arabidopsis* (Sarojam et al., 2010). And in tomatoes, the co-expression of *YABBY1* and *YABBY3* can regulate the leaf size both in cultivated and wild species, and the evolutionary analysis suggests that *YABBY1* emerged earlier than *YABBY3*, evolving from a common ancestor before the divergence of dicotyledonous plants into Rosids and Asterids (Filyushin et al., 2018). The down-regulation of *ARGONAUTE1* (*AGO1*) in tomatoes resulted in morphological defects in leaf adaxial-abaxial patterning, meanwhile, the expression level of some adaxial-abaxial domain formation-related genes also significantly changes, such as *ARF4* and *NPR5* (Wang et al., 2015). In tomatoes, driving the miR164-resistant form of GOB^m^ *via* the FIL promoter in tomatoes produced rounded, rumpled, and deeply lobed leaves (Berger et al., 2009). Besides, the ectopic expression of *HANABA TARANU* (*CsHAN1*) in cucumbers produced highly lobed leaves (Ding et al., 2015). The cooperation between these genes can determine the type of leaf margin.

## Summary and outlook

The previous studies on dicots *Arabidopsis* and monocots rice have revealed the formation process of leaf morphogenesis (Vercruysse et al., 2020; Wang et al., 2020), and provided further reference for the leaf morphogenesis of other plant species. Leaf morphology plays a vital role in some vegetable crops, especially, since leaves are the edible organs for some leafy vegetable crops including Chinese cabbage and lettuce. Therefore, the research on leaf morphology of vegetable crops can largely contribute to a high yield and quality which are the main goals of breeding. At present, MAS breeding has become a major method to improve breeding efficiency and shorten the breeding cycle. However, due to gene evolution and leaf characters, many genes showed new functions or mechanisms in vegetable crops that were species-specific. Understanding the molecular mechanism in different species also can provide more knowledge about leaf morphogenesis. This review summarized the identified genes and molecular mechanisms for leaf morphology in vegetable crops and provided a reference for the application of some reliable markers in vegetable crops. For example, heading is a typical trait for some leafy vegetable crops and there is always different demand for heading and non-heading cultivars according to the consumers’ preferences. Several heading-related markers (*BrpSPL9, BcpLH, BcpLH2*) have been identified in Chinese cabbage which can be applied for genotyping among different cultivars at an early seeding stage (Wang et al., 2014; Ren et al., 2018; Ren et al., 2020). Besides MAS, the transgenetic system has also become more reliable in some vegetable crops (Kim et al., 2021), thus, transformation by precisely regulating the target gene could be a possible approach for high-efficiency breeding. For instance, leaf size is often precisely controlled by the expression level of some leaf-size-related genes, including *TCP4*, *GRF*, and some microRNAs (Yu et al., 2013; Mao et al., 2014; Yang et al., 2018). The vector construction by overexpression, knockout, or knockdown of the target gene and transformation into certain cultivars can then largely proceed in the breeding process. With the improvement of genome information and the maturity of the genetic transformation system of various vegetable crops, the problems in transformation, such as the off-target and redundant functions of several genes, could be avoided as much as possible. Overall, due to the high consumption of vegetable crops, a reduced breeding cycle by screening at the seedling stage or genetic transformation could be conducive to breeding ideal cultivars for vegetable crops.

## Author contributions

NH and TW wrote the article. JC, CW, YZ and YD summarized the references. All authors contributed to the article and approved the submitted version.
